# The Anatomy of the Arteries of the Human Body, and Its Applications to Pathology and Operative Surgery; with a Series of Lithographic Drawings, the Size of Nature

**Published:** 1845-04

**Authors:** 


### Art. IX.

*The Anatomy of the Arteries of the Human Body, and its applications to Pathology and Operative Surgery; with a series of Lithographic Drawings, the size of nature.* By Richard Quain, f.r.s. Professor of Anatomy in University College, and Surgeon to University College Hospital. The Drawings from Nature and on Stone, by Joseph Maclise, Esq. Surgeon, in Eighty-seven Plates, imperial folio. The Letterpress 8vo, pp. 550.—*London,* 1844.

On the first appearance of this work, we announced its nature and design in our Number for January 1841 (Vol. XI), and foretold, from the character of the author, that it would both contribute to the anatomical knowledge of the vascular system, and bring out the facts required by the surgeon. In no respect has our prediction been unfulfilled; much that is important being now added to our previous knowledge, and much doubt and uncertainty removed. Nor has the artist failed in the execution of his part of the work. It is too apt to happen that artists fall off in execution as the novelty of their undertaking wears off; but in the present case the reverse has obtained; as it appears, from comparing the later with the earlier plates, that the beauty and accuracy of the workmanship have been throughout progressive. The work, as now completed, consists of a volume of folio plates, of the size of life for the most part, and an octavo volume of letter-press. Its nature will be best explained by the author's own statement of the plan of his work.

"The arteries are represented—1st, according to their most frequent arrangement, without the accompanying veins; 2dly, they are shown in connexion with the larger veins and the nerves; 3dly, the deviations from that which has been taken as the standard, because the most frequent, condition of the arteries are illustrated in a series of sketches; 4thly, such peculiarities of the veins, and occasionally of the nerves and muscles, as appeared likely to be of importance in surgical operations, are represented on a reduced scale.

"The letter-press, besides an explanation of the drawings and remarks on them, contains—1, a series of tables, showing, in a considerable number of cases, the condition of the arteries as to some of the points of most importance in their anatomy; 2, a connected view of their anatomical history—the details being arranged in systematic order; 3, practical commentaries; which consist, for the most part, of inferences from the facts previously set forth, and their application in performing surgical operations."

Although it would be supposed, on the slightest consideration, that a minute acquaintance with the blood-vessels and the deviations that are met with, both as to their course, branchings, and connexions, was of the greatest moment and necessary to every anatomist and surgeon, the subject has not hitherto received such attention as it merits. Until the time of the present publication it may be said that we knew with certainty but little of the variations in the ordinary disposition of the arteries, and their practical bearing. In this country the matter has been comparatively unstudied, and its consequence unrecognized. For in what treatise on surgery do we find enumerated with precision the unusual arrangement of the vessels and surrounding textures that may delay or complicate the operation of placing a ligature on an artery? It is true some attention has been given to it by Tiedemann, but the inquiry had not been followed out in its completeness, as in the present treatise on the anatomy and operative surgery of the arteries.

In an investigation apparently so endless as the variations of the blood-vessels, little value can be given to conclusions deduced from the examination of only a few bodies. As well might we endeavour to estimate the amount of rain in the year by the quantity falling in a given time in one shower, as attempt to fix the frequency of any particular arrangement of an artery by its presence in one or two bodies. The observations to be useful require to be continued during many years, and on a number of bodies sufficient to supply the average frequency. There are, therefore, but few surgeons who, with the opportunity, have the talent and application so necessary to come to a correct determination. Fortunately Mr. Quain possessed these requisites; and, from his position as Professor in the School of University College, he has had the opportunity of examining, more or less minutely, the anatomical disposition of the vessels in 1040 bodies. From such great experience it is but just to assert that our author is entitled to fix what is the standard conformation of the vessels, and to form "at the same time reasonable conclusions both as to the limits of the deviations from that standard, and the relative frequency of their occurrence."

For ourselves, we could find sufficient matter for praise in the fact of Professor Quain giving us an original work, though we think there may not be many who will participate altogether in our feelings; for in these days of utilitarianism any inquiry is undervalued unless the author is prepared to enunciate some practical application. This is especially the case in the study of the human body; the question of *cui bono* meeting the student of Nature at every step. But passing over the subject of the love of science for its own sake, we may inform our readers that the end aimed at by our author has been, altogether, the application to surgery of the information obtained. The inducement to inquiry arose from "the belief that the difficulties which have often occurred in the performance of those surgical operations in which the larger arteries are concerned, have arisen in great part from want of sufficient acquaintance with the differences in anatomical disposition to which these vessels are subject—not merely the deviations in the origin of large branches, which are usually named varieties, but other peculiarities of various kinds which are liable to occur, such as those which affect the length, position, or direction of the vessels."

In a book like the one before us, whose data are facts obtained by observation, there is little room for criticism; we can but record the information contained in it. We shall therefore select such parts as will illustrate the character of the work, and serve to impart some surgical knowledge. In the notice of any vessel, we shall state first what pertains to the unusual condition, and then consider the application of this to practice.

Arch of the aorta. The great systemic vessel of the body, from which the other arteries may be considered but offsets, is first considered by our author; and we cannot commence more judiciously than by following the same order. The commencement of the great trunk of the aorta presents a not uninteresting study to the physician seeking to detect disease, or to the physiologist who endeavours to explain deviations from the usual arrangement by reference to the persistent forms of the same part in animals lower in the scale than man. Professor Quain has not been unmindful of the aid comparative anatomy offers in the explanation of irregular appearances in the human body, and he accordingly attempts to illustrate some of the varieties in the disposition of the beginning of the aorta by examples obtained from the lower classes. We are accustomed to consider, because of its much greater frequency, the curvature of the aorta from the base of the heart to the left side of the vertebral column as the constant anatomical form, though the arching of the vessel to the opposite or the right side of the spine, so as to bend over the roof of the right lung, is occasionally seen. If we attempt to seek an explanation of this that appears at first sight strange, we discover that in the bird the aorta turns to the right, but in man to the left side; and that the different state is due to the persistence of one or another of the primitive branchial arches of the fetus. In an early period of the existence of the fetus of a vertebrated animal, the intestinal canal is surrounded near its front by five pair of vessels, which unite above the œsophagus in the trunk of the aorta; and from the obliteration or persistence of some of these result the different forms in the commencement of the aorta, and the pulmonary artery of the adult. These arches are more or less permanent in the vertebrated classes. Thus, in the cartilaginous fishes, the five pair of arches remain. In batrachia with tails only four are seen, the fifth or last being transformed into the pulmonary artery; in those without tails, as in the frog, only one branchial vessel is left on each side, constituting a bifurcated aorta, and the posterior arch is changed into the pulmonary artery. In birds the fourth arch on the right side remains as the aorta, whilst the last on the same side and the fourth on the left produce the pulmonary arteries. In mammals the aorta is developed from the fourth arch of the left side, and the pulmonary artery is derived from the last on the left and the fourth on the right side.

The few unusual changes of the trunk of the aorta, here described and represented, have been chiefly collected from the experience of predecessors; for our author says:

"The deviations from the ordinary disposition of the arch of the aorta referred to in the preceding pages, and represented in the majority of the figures in the fifth plate, are of very rare occurrence, insomuch that in the number of bodies (nearly a thousand) over which my observation has extended with a special view to the subject, no important changes were found in the arch, with the exception of those which occur in its position or height within the chest, and the single case of transposed aorta accompanying transposition of the viscera." (p. 42.)

The variations in the bend of the aorta refer either to change in position, or to unusual conformation.

*Change in the position.* The highest part of the bend of the arch, commonly placed rather less than an inch from the upper margin of the sternum, may vary from that spot opposite the interval between the first two dorsal vertebræ, to the middle of the fourth dorsal vertebra. The arch is ordinarily directed over the root of the left lung, though it may be bent in the opposite direction, to the right of the vertebral column as in birds. This deviation may be accompanied by transposition of the viscera and the other large vessels of the body, or, without any transposition, the aorta may take its accustomed position on the spine below the root of the right lung. From the rarity of the occurrence of the unusual position, surgeons may perhaps disregard it, though it is well to be informed of its occasional existence, inasmuch as the innominate trunk is then transferred from the right to the left side of the neck, and consequently the subclavian artery of the right side would be more difficult to find from its deeper position, and sharper curve over the first rib. But the relative height to which the arch reaches is of more consequence. If it is placed on a level with the fourth dorsal vertebra, the whole of the innominate artery, and the first part of the subclavian artery are contained in the thorax; so as to render almost impossible the ligature of either, and obscure the detection of an aneurism. On the other hand, if the arch extends nearly to the root of the neck, those same vessels will be accessible, and could be reached with comparative ease.

*Unusual conformation.* The instances reported of this state are both curious, and explicable by the permanent conditions in the lower classes. For the arch may be bifurcated as in reptiles, the œsophagus and trachea being contained in the inclosed space. Or the aorta may divide without an arch into an ascending and descending trunk as in quadrupeds.

"Hence the origin of the terms 'ascending' and 'descending' aorta. These words were introduced into anatomical language at a time when the systemic artery was supposed to divide, as shown in fig. 6, into two parts, which took opposite directions, and were properly named ascending and descending. The idea of the form of the aorta was then taken, as is generally known from its state in quadrupeds; but the terms were retained even when the error as to the fact had been removed." (p. 21.)

*Branches from the arch.* The branches of the aorta that ascend to the neck, and are concerned in operations, are the innominate,* left carotid, and left subclavian trunks, which spring from the top of the arch. The changes to which these are subject, it is not our intention to enter into in detail, though sufficient interest might be given to the subject by the statement of the illustrious Haller—that only the left vertebral artery had been seen by him to arise from the arch, even though he had dissected 400 bodies. Firstly, the origin of the three arteries may be moved nearer than common to the beginning of the aorta, or one may approach or join another. Secondly, the number may be diminished to two, the left carotid and innominate uniting, a variety found by Prof. Quain's observations to be the most frequent, though it is the general opinion of anatomists, that an increase in the number of the branches is most commonly present. Tiedemann gives in his work† a figure of this arrangement, and refers to Zagorsky who described it; but it appears that the accuracy of the drawing is very doubtful, for our author says with reference to the same;

"Now, on reference to the essay of the Russian anatomist, I find that he has not illustrated the description bya drawing; the representation given by Tiedemann is, in consequence, to be regarded as a plan made in conformity with the description in the original paper." (p. 46.)

Thirdly, the number three may remain the same, but the vessels be differently united. And fourthly, the number of the primary branches may be increased by the division into two of the trunk of the innominate, the four arising in a very varying order from right to left. Those who wish to pursue farther this subject, should consult the work, p. 47. It is, however, worthy of remark that when there are four trunks, the right

* We must thank our author for substituting English for Latin terms in numerous cases.

† Tabulæ arteriarum corporis humani, 1832.

subclavian is gradually transposed from the right to the left side, and arises in the greater number of instances altogether to the left of the others, so as to deviate apparently as much as possible from its usual origin. In those cases in which the arch is to the right side of the spine as in birds, the innominate trunk is destined to the left half of the neck, and separates into the left carotid and left subclavian; and when the arch is bifurcated, as in the instance described by Malacarne, the subclavian and the external and internal carotid of the same side of the body arose separately from the same arch.

Subclavian arteries. Both in an anatomical and a surgical view, there will be found matter that is new and exact in the portion of the work occupied with the consideration of the subclavian artery. In a vessel of such large size, hitherto so unsuccessfully ligatured, the attempt being attended with great loss of life, all facts in its anatomical history have a most momentous importance to the surgeon; for improvement in operative surgery depends upon the progress and knowledge of anatomy. Much of the want of success attending ligature of the commencement of the subclavian, is probably to be attributed to a lack of precision in the anatomy. Until the present inquiry much of our knowledge has been obtained from the examination of only few bodies, or, what is worse, from only general impressions instead of numerical precision. In illustration of the manner in which general statements are made, our author cites the instance of Dupuytren, who says that the subclavian is comparatively superficial in those persons with long and slender necks, but deep in those with short thick necks and muscular shoulders. Although Professor Quain is disposed to agree with Dupuytren, as to the influence of the shoulders, he gives, very justly, but little credence to the accuracy of the assertion, being conscious how often a general impression, when tested by observation, is found to be contrary to fact. He very properly observes:

"It should, however, be understood that my opinion as to the correctness of this statement is founded on the observation, with special reference to this point, of only a few cases, and on the general impression existing on my mind, and that it is not an inference from such a number of carefully examined bodies as would warrant entire reliance on its accuracy. There is no evidence that Dupuytren's statement has any better foundation." (p. 151.)

The part of this valuable work in which this important artery is described will give a fair idea of the information obtained by the researches of the author.

*Right subclavian.* Most commonly this artery is a branch of the bifurcation of the innominate trunk, and begins near the sternal end of the clavicle, the origin being above or below that spot according as the common trunk may reach into the neck or divide in the chest. Usually the two arteries (subclavian and carotid,) into which the innominate divides, are placed side by side, being equally far from the surface; but at other times the carotid is altogether in front of the subclavian. From its point of origin the subclavian courses outwards across the lower part of the neck, to the upper limb, forming an arch between the scaleni muscles, and giving branches upwards and downwards. The position of that arch when noted in twenty-five bodies was as follows: it was slightly (not half an inch) higher than the clavicle in eleven, and not higher than that bone in six instances; in the remaining eight it was higher in the neck, reaching in four cases from one inch to one inch and a half above the clavicle, so as to be for a longer extent in contact with the bag of the pleura. Our notice of the ordinary course of the vessel between the scaleni, is only for the purpose of drawing attention to the fact that it may deviate from that line. In a few instances the artery perforated the fibres of the anterior scalenus muscle, both with and without an accompanying trunk of the brachial plexus, and occasionally it has been found altogether in front of that muscle, in close contact with the subclavian vein. For the purpose of referring to the separate portions of the vessel, which vary in depth and consequently in the facility with which they may be reached, we may state that anatomists divide the artery into three parts, one internal to the scalenus, one beneath it, and one beyond that muscle. In the first part it is very deep, and has most complicated connexions; beneath the scalenus it is in contact with fewer nerves and veins, and is less deep; but in the third part it is most superficial, not being concealed by any muscle, though occasionally this portion may be covered by the trapezius extending along the clavicle so as to join the sterno-mastoid muscle, or by the omohyoid, (the posterior belly,) which may be so wide as entirely to reach over the vessel.

*Branches.* In estimating the circumstances that influence the success of the ligature of a large artery, such as the subclavian, it is impossible to disregard the number and position of the branches that are connected with it, seeing that they are the disturbers of the healing process set up by nature. "And,—considering the shortness of the trunk, the size of the offsets, and the manner of their arrangement on the parent vessel,—it may be confidently stated that there is no artery in which the influence alluded to is more considerable than in the subclavian." To ascertain the number and position of the branches on the trunk of the vessel in the three different parts of its course, in order to conclude what portion offered any chance of success in an operation, was made the subject of particular inquiry.

In the first part of the subclavian there are three branches, the vertebral, thyroid axis, and internal mammary, which are most frequently placed near the scalenus muscle, leaving thus an interval at the beginning unoccupied by branches. Observations were therefore made on sixty-five bodies, to determine how long was the interval available for ligature, between the origin from the innominate trunk and the branches, and with the following result:

"Length of the right subclavian artery from its origin to the point at which branches arise:

| | |
|---|---|
| ½ inch and under . . . | . 8 |
| more than ½ inch, and not exceeding 1 inch . | . 33 |
| more than 1 inch, and not exceeding 1½ inch | . 23 |
| 1¾ inch (the extreme length) . . | . 1" (p. 165.) |

In figure 5, plate 21, five branches are represented as connected to the part of the artery internal to the scalenus, covering the trunk, and not leaving any space for a ligature without encroaching on a branch.

A difference was observed in the second part of the subclavian on the opposite sides of the body; the absence of branches being most frequent on the left, and the presence of one most constant on the right side. By the abstract of the table it seems that in two thirds of the bodies examined, one branch, the superior intercostal, was present, and that in more than half the remainder there was not found any. Occasionally two, three, or more branches took origin from this part.

In the third part, where the artery is most frequently tied, and which is said in anatomical works to give but rarely a branch, our author finds sometimes one, two, three, and four branches. The abstract of the same table shows that only in about half the bodies observed there was not any offset—the proportion being 1 in $1\frac{6}{7}$; "and that in less than half—one example occurring in $2\frac{1}{6}$, it (the artery) gave origin to a single branch."

Having mentioned the facts that interest the surgeon, we may be excused if we consider shortly the details of some of the branches that concern the anatomist. Hitherto much confusion has existed in our works of anatomy respecting both the origin of the branches of the subclavian, and the names to be applied to them. The differences amongst authors have arisen in a great measure from the absence of data sufficiently numerous to decide which is the most frequent arrangement. Happily now we may consider the doubt resolved by the labours of Professor Quain, whose statement as to the most constant distribution must be considered an authority. And in future it must be inexcusable in any writer to disregard the information contained in the 'Anatomy of the Arteries, &c.' We should wish, did our space permit, to extract freely the facts of our author; nevertheless, we shall endeavour to collect such data as will display the extent of his researches, and oblige others to examine the work. We do not here intend the description of the arteries, only the points in which they differ from the common statement. In all treatises on anatomy it is said that five branches arise from the subclavian, viz. vertebral, thyroid axis, internal mammary, superior intercostal, and deep cervical. Our author fixes them differently, as vertebral, thyroid axis, internal mammary, superior intercostal, and frequently posterior scapular; this last arising beyond the scalenus in a ratio rather less than half of the number of bodies examined.

Firstly. The vertebral artery (*arteria vertebralis*) was not once seen to arise beneath or beyond the scalenus. It may be said to enter almost always the aperture in the transverse process of the sixth cervical vertebra, and very rarely that of the seventh. No example has been seen in which it entered higher than the third, though it is described as reaching as high as the second or first. Some curious instances of the origin of this branch by two or three roots have been met with, and an attempt is made to explain their source.

Secondly. The thyroid axis (*axis thyroideus*) springs from the first part of the subclavian, and divides into three branches, viz. inferior thyroid, transverse cervical, and supra-scapular.

The inferior thyroid (*art. thyroidea inferior*) gives as a branch the ascending cervical, which is sometimes described as a distinct artery from the thyroid axis.

The supra-scapular artery (*art. supra-scapularis*), lying across the bottom of the neck along the line of the clavicle, has the course and distribution commonly described.*

* This branch has been named *art. transversalis humeri;* but this term only adds to the confusion.

The transverse cervical artery (*art. transversalis colli*), a considerable trunk, crosses the side of the neck above the line of the supra-scapular, and divides beneath the trapezius into superficial cervical (*art. cervicalis superficialis*) for the muscles, and posterior scapular (*art. scapularis posterior*), directed along the base of the scapula. The above is the most frequent disposition of the transverse cervical, and may be said to be its usual distribution. But suppose, for the sake of explanation, that the artery should not be sufficiently large to separate into the two branches named, it ends entirely in the muscles on the side of the neck; and the posterior scapular artery will, in that case, spring as a separate branch from the trunk of the subclavian beyond the scalenus muscle. When noticing the origin of branches from the subclavian, mention was made of one being connected very frequently to the third part of the vessel; the branch then referred to is the posterior scapular, which is usually derived from the transverse cervical artery. It may seem needless, because the fact is so obvious, to direct attention to the influence that the so constant presence of a branch from the third part of the subclavian must have upon a ligature of the trunk; but it is our duty to remark that, before the present publication appeared, we did not possess any accurate knowledge concerning the frequency with which this arrangement might be expected. Our author has found three distinct modes of distribution of the transverse cervical artery, which are here enumerated in their order of frequency.

"(1) A considerable artery (transverse cervical—arteria transversalis colli) arises from the thyroid axis with the supra-scapular, and crosses the side of the neck at some distance above the clavicle and the last-named artery, as far as the levator anguli scapulæ, close to which muscle it divides into two principal branches. *a.* One of these (the superficial cervical—arteria cervicalis superficialis) runs superficially to the muscle just named, and ramifies under the trapezius, distributing branches to it and the muscles beneath it. *b.* The other branch (posterior scapular—arteria scapularis posterior) lies much more deeply than the preceding; it sinks under the levator anguli scapulæ, and turns downwards along the base of the scapula, beneath the rhomboid muscles. This disposition of the arteries existed in the body of which the second plate is a representation.

"(2) There are two separate arteries of nearly equal size. *a.* One arises, as in the preceding case, from the thyroid, and has the same course across the neck. It ends in branches under the trapezius muscle. From the similarity of the distribution of this artery to the superficial branch of the transverse cervical noticed in the foregoing paragraph, this is entitled to the same name (superficial cervical). *b.* The second artery usually arises from the third part of the subclavian, and, like the deep branch in the former case, is directed along the posterior margin of the scapula under the same muscles. This must obviously be named the posterior scapular artery. This mode of arrangement of the vessels is illustrated in the first and sixteenth plates.

"(3) In this, as in the second case, there are two arteries; but one is much smaller, and the other larger, than those which belong to that arrangement. *a.* The first artery is very slender. Arising from the thyroid, it has the usual course of the transverse cervical, and is expended in small ramifications at the side of the neck, between the sterno-cleido-mastoid and the trapezius muscles. This might be considered to correspond to a part of the superficial cervical. *b.* An artery of considerable size, given from the third part of the subclavian, divides opposite the levator anguli scapulæ into two divisions. One of these supplies the trapezius with the muscles which it covers, and may be regarded as corresponding to the greater part of the superficial cervical of the other cases. The other division of the artery has the usual course of the posterior scapular. The disposition of the arteries here pointed out was present in the body from which the third and the nineteenth plates were drawn—the size, the division, and the arrangement of the vessels are illustrated in the latter plate." (pp. 175-6.)

Thirdly. The superior intercostal (*art. intercostalis superior*) and deep cervical (*art. profunda cervicis*) have been ascertained by a great number of observations (266 out of 285 instances) to arise by a common trunk beneath the anterior scalenus muscle. In our text-books of anatomy the two are described as distinct branches with separate origins. From the almost constant occurrence of this common origin, it seems difficult to account for their being disunited ; and it is a good example of the manner in which one writer follows another in continuing an error. In consequence of the constancy with which the two are joined, our author considers the artery named deep cervical as only a posterior or muscular branch of the superior intercostal, analogous to the posterior offset from every other of the intercostal arteries of the aorta. Consequently it may be omitted from the primary branches of the subclavian. In its course backwards this branch (deep cervical) is placed between the transverse processes of the last cervical and first dorsal vertebræ.

Fourthly. In addition to the branches usually described, a small artery (? spinal) arises from the subclavian beneath the scalenus, and is distributed to the nerves of the brachial plexus, and the interior of the spinal canal.

*Right subclavian from the arch of the aorta.* Besides the common place of origin opposite the sterno-clavicular articulation, the right subclavian takes origin, in those cases in which there is not any innominate trunk, directly from the arch, either preceding the other branches from this part, alternating with one or other, or following them. Of all the variations, that in which it is the last branch is the most frequent, and has been seen four times by our author. With this last origin the vessel crosses obliquely the spinal column, beneath the trachea and œsophagus, and takes its accustomed place between the scaleni muscles. It must be obvious, therefore, that should it be necessary to ligature the vessel, its position would be deeper and lower in the neck than when it comes from the innominate artery. When the artery has the position behind the œsophagus, it may be injured by force acting on the œsophagus, or by a foreign pointed body impacted in the gullet. No difficulty of swallowing during life has been traced to the existence of the subclavian in that position, in the instances in which after death this variety has been found. If, however, the aberrant artery lies between the trachea and œsophagus, as in a case published by Dr. Bayford,* there is difficulty of swallowing; and in the case referred to, this increased in violence at puberty, augmented in intensity periodically or during exercise, and lasted till death at sixty-two years.

It is a remarkable circumstance connected with this variety that, in the examples of the origin of the right subclavian from the left side of the arch, the recurrent nerve of the right side arose from the pneumogastric opposite the lower part of the larynx. Dr. Hart has attempted to explain this departure from the regular order by reference to the near position of the larynx and arch of the aorta in the fetus.† He says:

* Memoirs of the Medical Society of London, vol. ii, 1793.

† Edinb. Med. and Surg. Journal, vol. xxv, p. 287.

"In the earlier periods of the existence of the fetus, the rudiment of the head appears as a small projection from the upper and anterior part of the trunk, the neck not being yet developed. The larynx at this time is placed behind the ascending portion of the arch of the aorta; while the brain, as it then exists, is situated so low as to rest on the thymus gland and front of that vessel. Hence it is that the inferior laryngeal nerves pass back to the larynx, separated by the ascending aorta, the left going through its arch, while the right goes below the *arteria innominata.* As gestation advances the head becomes more distinct, and the neck begins to be formed after the second month, which, as it lengthens, has the effect of removing the brain upwards to a greater distance, and of drawing out the larynx from the chest, in accommodation to which the nerves of the *par vagum* and their recurrents become elongated, and hence the circuitous route the latter are found to take afterwards, forming loops in which the aorta and right subclavian artery are, as it were, suspended."

*Left subclavian from the arch.* This is the regular arrangement in bodies without variety in the arch. But in those bodies in which the arch bends to the right side of the spine, the left subclavian would be produced commonly by the bifurcation of the innominate which is then directed to the left half of the neck. If the innominate is wanting in that peculiar disposition of the aorta, the subclavian may commence last on the arch, and will therefore cross the spinal column, from right to left, behind the trachea and œsophagus. Should there be transposition of the viscera and pulmonary artery, as well as of the arch, in those instances in which the subclavian arises last, this vessel has no further peculiarity. But should there not be the transposition of the pulmonary artery with that of the arch, the pulmonary artery still remaining on the left of the aorta, it is evident that the canalis arteriosus that joins those two parts in the fetus, must take a circuitous course behind the œsophagus and trachea to reach its destined point; in such a case the left subclavian, not arising from the innominate, is found connected with a pouch or dilatation. The presence of this pouch has been remarked by the narrators of different cases, but no attempt had been made to explain its nature. Professor Quain has carefully examined the examples of the origin of the left subclavian from a pouch in both the fetus and the adult, and tracing the dilatation through successive stages has concluded with great sagacity, that the "pouch" is but a portion of the canalis arteriosus, or tube uniting the pulmonary artery with the aorta in the fetus, that remains unobliterated through life in consequence of the left subclavian artery arising from it. So the ductus arteriosus will be ligamentous only in part, the upper end remaining pervious to blood for a short distance, and constituting a portion of the trunk of the subclavian, in the same way as the branches of the umbilical vein to the left lobe of the liver in the fetus, remain open and become part of the left division of the vena portæ of the adult. As we could not condense, without destroying its character, the description of the steps by which the author has come to that determination, we are compelled to extract it entire notwithstanding its length.

"In order to explain the conclusion I have arrived at, as to the nature of the pouch, for such it may be named for the sake of convenience, it will be necessary to give a statement of the facts that have led to the conclusion. These may be arranged into two sets: according to the position which the pulmonary artery and the ligamentum arteriosum or canalis arteriosus bear with reference to the arch of the aorta.

"*a.* The vessels having their usual course and position: a small nipple-shaped dilatation is not unfrequently observable on the concave side of the arch towards the left side, projecting at the point at which the ligamentum arteriosum is united to the aorta. The connexion of this dilatation with the ligament just named, suggests that it is an unobliterated part of the canalis arteriosus. But the dilatation from which the subclavian artery has been found to arise, is of considerably larger size than those now noticed, (see plate 7, fig. 2, b.) In accounting for this fact it will be assumed for the present that the subclavian artery may arise from the canalis arteriosus near its end. Now, supposing this to be the case, it is manifest that, blood being transmitted from the aorta into the subclavian artery through the end of the canalis arteriosus, the obliteration of the upper part of this tube must, as a necessary consequence, be prevented. The greater size of the pouch is, then, according to the view here taken of the subject, owing to the origin of the large artery from it. It may here be added, that should the aorta be directed to the right side, the change of its course being accompanied by the general transposition of the viscera, the pulmonary artery undergoes a corresponding alteration of its position; it is placed on the right side, and the ligamentum arteriosum joins the concavity of the arch in the usual way, (plate 5, fig. 4.) And, moreover, when the aorta has the unusual direction, at the same time that the viscera retain their ordinary position, the course of the pulmonary artery and the ligamentum arteriosum may be, but, as will presently appear, is not always the same as that just indicated. This fact is illustrated in the body of a fetus described by Mr. Abernethy, and noticed in a former part of this work, page 18, and plate 5, fig. 5. From the circumstances before stated it is apparent that, as the canalis arteriosus joins the arch of the aorta in the ordinary way in these various cases, a dilatation, if any existed, would probably be the same as those already described.

"*b*. A very different disposition from the preceding exists in the two preparations represented in plate 7, fig. 1, and plate 20, figs. 8 and 9: for while the aorta arches to the right side, the pulmonary artery retains its usual position on the left, and the ligamentum arteriosum joins a pouch projecting from the aorta. Now, an inspection of the figure (8) will show that the canalis arteriosus, in order to join the aorta at the end of its arch, must, in such a case as this, necessarily pursue a circuitous course, and cross the trachea and œsophagus. If it had the transverse course behind these tubes, if also the subclavian artery arose from it, the appearance of the vessels would be easily explained, for the pouch would be in these, as in the former instances, the end of the arterial canal, unobliterated, because of the transmission of blood through it from the aorta.

"In order to complete the view here presented of the nature of the dilatations or pouches under discussion, it is necessary to show the left subclavian artery arising from the canalis arteriosus in the fetal state before any part of this canal has been changed into a ligament. Cases in which the subclavian artery and the 'arterial canal' had this disposition, are recorded by Klinkosch and Meckel, and two figures contained in the essay of the former are copied in plate 20, figs. 10 and 11. In one of the figures the heart and large vessels are viewed from before; in the other, the parts are seen on their posterior aspect. The aorta and pulmonary artery have, as they arise from the base of the heart, their ordinary relative position, (see fig. 10;) the former is directed to the right, and the latter to the left side of the trachea and œsophagus. On an inspection of the 11th figure, the vessels being viewed from behind, the canalis arteriosus (14) is seen to have a transverse course behind the œsophagus and the trachea (*d*) and (*h*), and to join the aorta; and the left subclavian artery (5′) is derived from it at some distance from the aorta. In conclusion: supposing the case last mentioned to undergo the usual changes which occur after birth, a doubt cannot be entertained that it would be brought into the exact condition of those referred to immediately before; for the canalis arteriosus would be partly reduced to the state of a cord or ligament, but this alteration would not extend beyond the point at which the subclavian artery takes its origin; and the remainder, transmitting blood from the aorta into the artery just named, and being larger than the vessel given from it, would constitute a pouch." (p. 158.)

*Ligature of the subclavian.* After our extended notice of the facts collected by our author on the anatomy and varieties of the subclavian arteries, let us bring the additional knowledge to bear on the operation of ligature of those vessels. Since the period of the first application of a ligature to an artery to obliterate an aneurismal tumour by diminishing the flow of blood through it, the subclavian, both from its size, its branches, and the number of important parts in connexion with it, has obtained much attention from surgeons. Anything that may bring certainty into our practice ought to be most eagerly received; and surgeons should carefully examine this work, in which are distinctly enumerated the different circumstances that may render ligature of the subclavian more difficult, or diminish the chances of a successful operation.

The subclavian may be tied beyond the scalenus muscle for aneurism of the axillary, or upper part of the brachial artery. As before stated, the vessel is found most deeply placed in the first part of its course and most superficially in the third, consequently, whilst the ligature of the former is extremely difficult, that of the latter is comparatively easy. The operation is made close above the clavicle, or between this bone and the omo-hyoid muscle, the incision being limited in front by the sterno-mastoid muscle. The proceeding is not generally difficult unless the parts have been altered or displaced by disease, or its progress is impeded by any of the following uncommon dispositions of the vessels and muscles. In the first incision the external jugular may be wounded in consequence of either its enlarged size or unusual position, and to avoid it the integuments are divided on the clavicle; but it should be remembered that the external jugular may cross over the clavicle to reach the subclavian vein, or the cephalic vein, or a branch from this may ascend over the same bone to join the external jugular. The depth from the surface is increased in persons with short thick necks, high clavicles and prominent shoulders, but more particularly by the carrying up of the limb as an effect of a large aneurism in the axillary space. The surgeon may be delayed in the operation by finding the sterno-mastoid and trapezius muscles united along the clavicle, thus covering entirely the interval in which the artery lies, or by the omo-hyoid (the posterior belly) extending completely over the artery, instead of forming only the outer boundary of the space. The line of direction of the trunk of the artery may be much changed. Commonly it is a little above the clavicle, when the tubercle on the first rib, at the insertion of the anterior scalenus, will serve as the guide to the vessel. As the vessel is almost altogether on the rib, there is not much danger, with moderate care, of wounding the pleura. It may, however, be more superficial, passing through the fibres of the anterior scalenus, or even in front of it. Occasionally the artery arches much higher into the neck, being one inch and a half above the clavicle, and higher than this bone can be raised by the elevation of the shoulder. In this last condition the first rib cannot be any index to the place of the artery, which will be found by tracing upwards the posterior edge of the scalenus muscle, and by taking advantage of the large nerves directed downwards to the subclavian from beneath the muscle. Of the greatest consequence too, in the high arch of the vessel, is the state of the pleura, for all the third part of the artery is in close contact with the bag of the pleura that rises very high into the neck, and may very readily be punctured. Supposing the artery found, the securing it may be delayed by the subclavian vein, that may be placed on a level with the artery, or even conceal it, and is therefore in danger of being transfixed and included in a ligature, as was done by M. Robert. In nearly half the bodies observed, one branch of artery was connected to the third part of the subclavian, arising near the scalenus muscle; but as the result of ligature of the vessel has been so successful this does not appear to interrupt the progress of cure, or it may possibly have been absent, or sufficiently far from the ligature not to interfere with its action. Such then are the principal circumstances that may interfere with the operation of ligature of the subclavian beyond the scalenus, but with some caution and a due degree of dexterity they may be mastered.

It has been thought necessary to tie the first part of the subclavian for aneurism affecting the vessel beyond the scalenus. Besides the depth of this part of the trunk of the vessel and the difficulty in reaching it, circumstances common to it with other vessels, the branches have here the greatest influence in preventing the formation of a clot; for as before mentioned this part of the artery is thickly studded with offsets, so that only about one inch of the trunk, in the most favorable cases, intervenes between them and the origin from the innominate, and oftentimes there is not any interval sufficient for tying the vessel with a prospect of success. The contemplation alone of the number and position of the branches, and the nearness of the large carotid trunk to a ligature placed on the subclavian internal to the scalenus, might lead an anatomist to infer that hemorrhage would ensue on the ulceration of the coats of the vessel; and the practice of surgeons fully corroborates the truth of the inference, for death has followed the application of the ligature. To cut off the disturbing influence of the rush of blood in the carotid, Professor Quain proposed to tie both the carotid and subclavian trunks near their commencement. The operation has been tried by Mr. Liston, but without success. The same distinguished surgeon wished to repeat this new operation in another case of aneurism of the third part of the subclavian, but in the progress of the operation the trunk of the subclavian was found so much deeper than common, that it was supposed the vessel arose as a separate trunk from the arch, and therefore only the subclavian was included in the ligature. On examining the body after death, the subclavian was found to come from the innominate, but it took rise altogether behind the carotid, and was separated from this by a layer of fascia. In any repetition therefore of this operation, before it is concluded that, because the subclavian is deeper than usual, it must come as a distinct trunk from the arch of the aorta, the operator should compress the innominate trunk, or at least the origin of the carotid, to see if the flow of blood is thereby diminished. If then the result of the attempt to ligature the subclavian internal to the scalenus seems opposed to farther trial, still less justifiable would it be to endeavour to tie the corresponding part of the left subclavian, concerning which the opinion of our author is strongly expressed in the following passage:

"The left subclavian being nearly vertical in direction from the arch of the aorta to the scalenus muscle, only a small part can be said to be placed to the inner side of the scalenus after its emergence from the thorax; and the entire of the artery from the aorta to the muscle is so deeply placed, and, at the same time, covered by such important parts, which do not admit of being moved aside—namely, the pleura and the lung with the internal jugular and innominate veins—that no operation has been hitherto undertaken on this division of the vessel; and indeed an effort to expose it in a surgical operation does not seem admissible." (p. 165.)

Our full notice of the subclavian arteries will be sufficient to illustrate the practical nature of the knowledge presented to us, and will excuse further remark on the vessels of the head or trunk. Were we to notice all that is new, we should be compelled to give an analysis of most of the work. In this difficulty we shall select the trunk of the chief artery of both the upper and the lower limb—vessels that often require surgical interference in consequence of disease or injury at a distant point.

Brachial artery. The examination of the vessels of the limbs shows, as might be anticipated, that the farther the leading artery recedes from the trunk of the body the more the constancy of arrangement is departed from. Even in the large brachial artery of the upper arm an early division is a very frequent occurrence. Commonly the vessel bifurcates into radial and ulnar branches rather below the elbow-joint, but it may divide into two in the axillary space or at any point intermediate between this and the usual place of termination. The frequency of the early division was specially examined in a certain number of bodies and with the following result. In 481 bodies the vessel was found divided in 94 instances, thus giving two large trunks in the upper arm in about 1 in every 5 bodies. It has long been doubtful whether there exists a correspondence between the opposite limbs of the same body, that is to say, whether a usual or an unusual disposition of the vessels of one limb is accompanied by a similar one in the same part of the opposite side of the body. Bichat asserted that there is not any correspondence, but Meckel states that the same distribution, whether regular or irregular, occurs in both limbs, in accordance with an opinion that "the lateral symmetry, the most powerful of all, is maintained even in malformations." Tiedemann observes that the high division of the humeral is frequently found in both arms, and further adds a statement, which we do not think will be found exact—viz., that chiefly men of small stature have this variety.* The question cannot be decided by either of those writers, because neither has examined a sufficient number of bodies. Professor Quain has given his attention to the same subject, and has observed sufficiently extensively we think to have some weight in determining what degree of correspondence exists between both arms. The conclusion he comes to is, "that most commonly there is not a correspondence between both arms with respect to the high division of their arteries." He extracts from his observations the number of instances of the high division of the artery of the upper arm, examined in both limbs of the same body. The number so noted was 61, of which the following is the particular distribution:

*"Haüfig kommt die hohe Theilung der Armarterie an beiden Armen vor, wie Heister Petsche, Monro, Meckel, und andere richtig bemerkt haben. Ich sah diese Anordnung öfters. Besonders sind Menschen von kleiner statur dieser Abweichung entworfen."

| | | |
|---|---|---|
| " The entire number of cases is.................... | | 61 |
| The high division existed on both sides in the same position, in.................................. | 5 | |
| It occurred on both sides but in different positions, in.. | 13 | |
| | —18 | |
| It occurred on but one side, the artery on the opposite side dividing at the usual situation, in......... ... | 43 | |
| | —61" (p. 267.) | |

When the trunk of the brachial takes its accustomed course in the upper arm, it is along the inner border of the biceps muscle with the median nerve. But every anatomist and surgeon must be aware that the vessel is often removed from its regular position, and that in consequence any one ignorant of the fact might fail in an attempt to find it. The course of the artery, when not at the edge of the biceps, is along the internal intermuscular septum to the inner condyle of the humerus, where it is covered by the fleshy fibres of the pronator teres muscle, which is always in that case wider than common. The median nerve follows the deviating vessel. Sometimes the errant trunk of the artery winds round a projecting portion of bone on the lower third of the humerus, or it may be covered by a ligamentous band from the same portion of bone, which bounds an aperture resembling the foramen through which the humeral artery turns in the lion. The brachial, as before remarked, may be divided into two as high as the axilla, thus giving two large trunks in the position of the one; and the extent for which two vessels are found will depend upon the place of bifurcation. Either of the three arteries of the fore arm,—viz., radial, ulnar, and interosseous, may have a high origin, but the radial most frequently, then the ulnar, and lastly the interosseous. Notwithstanding the apparent irregularity with respect to the division of the brachial, our author never found more than two arteries in the upper arm below the bifurcation, except in one case in which all the three arteries of the forearm sprang together a little above the condyles of the humerus. When two arteries occupy the upper arm they are generally close together at the border of the biceps, the radial branch being most superficial; but of great importance is it to the surgeon to know that the larger trunk—the continuation of the brachial as it divides into ulnar and interosseous—may leave the edge of the biceps, may be directed along the internal intermuscular septum to the inner condyle of the humerus, and then be covered by the pronator teres as exemplified in the brachial trunk. The median nerve will accompany the larger or brachial division. The arteries, in the instances of high division, may be so largely connected together at or near the elbow as that the blood, in case of ligature of only one, would flow almost as uninterruptedly along the limb as if no attempt had been made to arrest it. For a specification of these different states the work must be consulted. In connexion with the union between the vessels, it may be remarked that the trunk of the humeral is sometimes bifurcated for a short distance, the portions however soon reuniting; and that small slender arteries, "*vasa aberrantia,*" spring from the humeral, and join one of the arteries of the forearm, most frequently the radial. Certain peculiarities, or modifications in form of the muscles and nerves, may complicate the surgical anatomy of the brachial artery. Thus the vessel may be covered by a muscular process from the biceps to the internal intermuscular septum, or by a third origin to the biceps from the humerus. And in the instances of high origin of the radial, the other division—the trunk for the remaining arteries of the forearm—may be entirely covered to a greater or less extent by a muscular layer of the brachialis anticus. Of the accompanying nerve, the median, there is but little to notice; it may be over or under the mid part of the artery, or it may have two or more cross branches over the vessel.

As a caution in venesection, it may be well to refer to the position that the arteries of the forearm, in the instances of high origin, may have to the veins at the bend of the elbow. Both the radial and the brachial trunk may be side by side beneath the median basilic vein, separated only by the aponeurosis of the arm; or the radial may cross beneath the same vein, above the aponeurosis and in contact with the skin. When the ulnar has a high origin, it gradually inclines inwards to the elbow-joint from the line of the brachial. The author observes; "The origin being above the ordinary position, the vessel is almost invariably situated over the superficial muscles of the forearm. With so much constancy is it thus placed that I have never in more than a single example (plate 36, fig. 2) found the artery covered by the muscles. Most commonly the ulnar artery lies beneath the fascia, between it and the muscles, as represented in plate 32, figs. 1, 2, and 3. Only three instances have occurred under my observation in which it was altogether subcutaneous." With the position either beneath the aponeurosis or above it, the ulnar crosses beneath the median basilic vein, and is liable to injury. The mere mention of the different varieties in the situation of the arteries coursing to the forearm will be sufficient to convince all of the necessity of well examining the bend of the elbow before thrusting a lancet into a supposed vein. The position of the vessels at the bend of the elbow is well and strikingly represented in plate 41.

*Ligature of the brachial artery.* It is manifestly the duty of every surgeon who has not had opportunities of extended personal observation, to make himself familiar with the facts collected in the works of others, in order that, when about to place a ligature on the principal vessel of a limb, he may feel conscientiously prepared for any known irregularity that may chance to present itself. To a reflecting man the placing the life of an individual in jeopardy by an operation is a sufficient responsibility, and an unexpected state of the structures concerned must give great embarrassment, especially if this arises from want of inquiry. In the common operation of tying the brachial in the middle of the upper arm, the proceeding may be, so to speak, comparatively easy, if all the parts have their common position. But the surgeon may be interrupted at the outset by finding a large muscular slip of the biceps between the knife and vessel when the aponeurosis is divided, instead of the arterial trunk and the median nerve. Again, the artery may be absent from the border of the biceps, and may be placed along the internal intermuscular septum, where it should be sought if there is not any trunk near the biceps. It has been further said that frequently, about one in five, there are two arterial trunks in the upper arm, in consequence of a high division. When such a state exists it may be necessary, in hemorrhage from the hand that resists all local attempts to stop it, to arrest by ligature the flow of blood in the limb. Here, supposing the surgeon to attempt the ligature of the brachial at the common place, the rule of compressing the supposed vessel before tightening the thread, to see whether the bleeding is commanded by this manual proceeding, is most imperatively necessary; for one or both vessels may be required to be ligatured before the bleeding ceases. The surgeon, however, may make his incision at the edge of the biceps and find but one artery (the radial), which, when pressed, has little or no effect upon the bleeding part. He will immediately presume that there is a second trunk, but unless he is aware of the occasional departure from the common anatomical arrangement he will not find it. With the information now collected for him he will know that the second trunk, if not close to the other, will be found either along the inner intermuscular septum, viz. in a line to the inner condyle of the humerus, or near its usual situation, but deeply placed, and covered by fibres of the brachialis anticus muscle. Further, in a wound at the lower part of the upper arm, where the two vessels exist, the one (the radial) by the side of the biceps would be seen and secured, but the larger and deeper (the continuation of the brachial) covered by the muscular fibres might retract, and not be thought of or seen at first, though its pouring out of blood afterwards, at intervals, would endanger life, and puzzle the surgeon ignorant of the peculiarity here specified.

THE FEMORAL ARTERY. The leading vessel that supplies blood to the lower limb merits the attention of the surgeon as much as that of the arm. Like the vessel of the upper limb, it remains undivided till it reaches rather below the joint (the knee), and then separates into branches for the leg. In the arm, the transposition of one of the branches of division from its usual place of origin to some point nearer the trunk of the body, is very frequent; but in the lower limb there is not any authentic notice of the branches of bifurcation of the femoral, viz. the anterior and posterior tibial, arising higher in the limb than the back of the knee-joint. Though the large terminal divisions are not transferred occasionally to the great trunk, the muscular branch or branches of the thigh so vary in the position of their origin as to influence greatly the result of the application of a ligature to the main trunk. The position of the varying branches, and the means by which they may be avoided, we shall next review.

The position of the femoral artery may be stated in general terms to be oblique in the front of the thigh, being placed in the hollow between the adductor and extensor muscles. For the first four inches it is uncovered by muscle, and is easily reached, but lower down it is concealed by the sartorius. Commonly the femoral extends as an undivided trunk from Poupart's ligament to the aperture in the adductor magnus, but it may be split in a part of its length, constituting a double femoral, similar to the condition occasionally met with in the brachial artery. This division may reach from a little below the large muscular branch, the profunda, to near the opening in the adductor, leaving the trunk single above and below those points. The peculiarity here referred to is so rare, that it was observed by our author but once in nearly 1200 bodies, yet as its existence created some difficulty in an operation for popliteal aneurism by Sir C. Bell, it will receive special consideration under ligature of the femoral. In the large vein that accompanies the femoral artery there are some variable conditions. In the regular order it winds behind the artery, being internal to this on the pubes, and external to the same beneath the sartorius. Instead of the vein being a single trunk, it may be divided into two, one being on each side of the artery, or one of these may cross the femoral, or the two be united by a series of small loops over the artery. The only muscle that has an irregular position to the artery is the sartorius. As before remarked, the femoral is uncovered by muscle for four inches, except when the direction of the sartorius across the limb is higher than common, for it may be so altered as not to leave two inches of space between the upper border and Poupart's ligament. This extent upwards of the muscle is mostly due to increased size, but Professor Quain supposes also that it may depend on the circumstance of the crest of the iliac bone being more curved inwards.

*Branches.* The chief interest connected with the branches of the femoral is the position of the large profunda, which determines the spot at which the parent trunk may be secured with the greatest prospect of success. This branch is the muscular offset of the thigh, and the anastomotic channel by which the circulation is carried on when the course of the blood is stopped in the main trunk; from it is given outwardly a branch (external circumflex), internally a second (internal circumflex), and the continuation, placed beneath the trunk of the femoral, is consumed in muscular and perforating branches. The profunda (deep femoral), or the external or internal circumflex branch of it, may be fixed to almost any part of the trunk of the femoral before this is covered by the sartorius, as stated by our author:

"With the view of ascertaining the point with some degree of accuracy, the distance between the place of origin and the lower margin of Poupart's ligament (which was taken as the line of demarcation between the iliac and the femoral parts of the arterial trunk) was measured in several cases. The details of this examination are contained in the table (page 475 et seq.), and the following is an abstract of it:

| | |
|---|---|
| The entire number noted in the table . . . . . | 431 |
| The deep femoral arose | |
| above Poupart's ligament, from the ex. iliac (plate 72, fig. 3) . | 1 |
| under that structure (plate 72, fig. 4) . . . | 7 |
| below it, ½ inch and less (plate 73, figs. 2 and 3) . . | 13 |
| more than ½, not exceeding 1 inch (plate 74, fig. 5), those measuring 1 inch, being 65 of the number . | 86 |
| more than 1, not exceeding 1½ (plate 73, figs. 1, 4, 5, plate 74, fig. 4 . . . . | 183 |
| more than 1½, not exceeding 2 (plate 72, fig. 6, pl. 74, fig. 3) | 109 |
| more than 2, not exceeding 2½ (plate 73, fig. 6) . . | 19 |
| more than 2, not exceeding 3 . . . . | 12 |
| 4 inches (plate 72, fig. 5) . . . . | 1 |
| | 431 |

"Thus, in about three fourths of all the cases (351 in 431), the origin of the deep femoral was from one inch to two inches, both these points included, distant from Poupart's ligament; and this may be said to be its usual position." (p. 520.)

If the origin of the profunda exceeds the distance of two inches from Poupart's ligament, the branch has a tendency to be resolved into the three portions before named, which may arise separately from the femoral trunk. In these instances the circumflex arteries spring somewhere near the parts to which they are distributed, so as to avoid, thus to speak, a backward course to their destination. In the last instance referred to in the extract, and represented in pl. 72, fig. 5, this disposition is exemplified; the internal circumflex arising half an inch below Poupart's ligament, the external circumflex about two inches, and the third portion for the perforating arteries ("profunda"?) four inches below the same part. From the observations made it appears, that no point of the femoral within four inches of Poupart's ligament should be selected for the application of a ligature, in consequence of the variations in the origin, either of the profunda or one of its branches.

*Ligature of the femoral.* This operation has been practised in two spots; the one near to Poupart's ligament above the origin of the profunda, the other where the vessel is about to be covered by the sartorius. The propriety of choosing the one or the other, according as each may be favoured or opposed by the facts set forth by our author, it is now our purpose to examine.

It has been stated that, between the line of Poupart's ligament and the origin of the profunda, the femoral measured, in 292 instances out of 431 noted in the table, only from one to two inches, and that in 183 of these it was not longer than one inch and a half. If it be further remembered that two large branches, the epigastric and circumflex, arise from the commencement, it must appear that the spot near Poupart's ligament is unfavorable for applying a ligature; for to whatever portion of this part of the femoral trunk the thread is applied, it will, in the majority of cases, be too near to branches either above or below to permit the healing process to go on undisturbed. Add to this, that the part of the trunk available for the operation may be shortened by the origin of the profunda being placed only half an inch or less from the ligament, by the transfer to it of branches of the profunda,* or by the epigastric and circumflex iliac descending rather below their usual origin, and it will be manifest that the knowledge obtained from the anatomy of the vessels would be opposed to the probability of ligaturing this portion of the femoral with success. Experience, should we not say too dearly bought, has conducted surgeons to a similar conclusion; ligature of the external iliac artery, or death from hemorrhage being the common consequence of the operation. In a wound of this part of the vessel it would be proper to secure the wounded part; but in other circumstances, such as hemorrhage from the lower part of the limb, or from a sloughing stump where the main artery cannot be recognized, for which this portion has been formerly tied, a surgeon would not be justified in proposing or attempting to place a ligature above the origin of the profunda.

The other spot in which the femoral is selected for the application of the ligature, is after it has given origin to the profunda and is about to be covered by the sartorius. The operation performed here is that of Scarpa, and is a modification of the one of Hunter, lower down in the thigh. Without discussing the merits of a particular proceeding, let us consider what anatomical circumstances may interfere with the obliteration of the vessel, or retard the progress of the surgeon. From the table it appears that the profunda arose more than two inches below Poupart's

* Some curious transplantations of the internal circumflex to the contiguous branches are represented in plate 74.

ligament in thirty-two bodies, and that in one it was found four inches from that structure. Therefore, as a branch or branches of sufficient size to interrupt the process of obliteration may be found three or four inches down the trunk, the ligature should be placed below their influence, or four to five inches from the ligament. In the first incision of the operation, the surgeon is to guard against a branch of the great saphenous vein crossing from the outer side of the thigh. Next, the sartorius muscle may entirely conceal the vessel, for it sometimes crosses the limb within two inches of the ligament, and "its position is such, that even above the middle of the thigh it appears more easy to expose the artery by turning the sartorius inwards than in the opposite direction." When the sheath of the artery is opened the vein should be, commonly, behind or to the outer side, but there may be two, as *venæ comites*, with or without communicating branches over the artery; these varieties in the veins cannot be disregarded, for they add greatly to the difficulty and danger of the operation. Finally, the operator may find the femoral trunk split into two of nearly equal size; these would be separated by a process of fascia, and join above the opening in the adductor muscle. In such a distribution what would be the practice to adopt? Such a conformation of the vessel was met with by Sir C. Bell, when tying the femoral for popliteal aneurism. At the time of the operation two vessels were not recognized, but the operator was perplexed by finding the pulsation return, nearly as distinctly as before, in a few seconds after the thread was tightened. A suspicion arose that the femoral was not included in the ligature, but Bell was certain he had tied the vessel, and resolved to wait the result. The death of the patient explained the mystery, and showed two femoral arteries instead of one. At that period the occasional existence of two arterial trunks was not known, and the surgeon could not do more. But now it is the duty of the surgeon to ascertain, before drawing the ligature on the coats of the vessel, that the pulsation can be made to cease in the tumour. Should the throbbing continue, notwithstanding the constriction on the one, the operator should divide the fascia, and look for a second trunk beneath or near the one found, in order that it may be also secured. The surgeon would most probably have some suspicion of the existence of this variety in the vessel by finding, as did Sir C. Bell, that pressure near Poupart's ligament stopped the pulsation in the tumour, but that compression lower in the thigh made no difference in either its beat or size, the finger in the former spot being above, and in the latter below the point of bifurcation.

The preceding analysis of a part of Professor Quain's work we offer to our readers as an inducement to farther examination. It is now our duty to inquire in what respect this work differs from those that have preceded it, and to assign to it its place in the rank of publications on medical science.

The writings with which Mr. Quain's work may be more especially compared are those of Haller, Scarpa, and Tiedemann. The volumes of the two former authors differ altogether; and their purpose has been expressed with much truth and candour by our author in his preface. The work of Tiedemann approaches much nearer in design, as it represents in a series of lithographic plates the arteries of the body according to their natural size, position, and course, so as to serve, as the author expresses it, for the study of anatomy, medicine, and surgery. This work contains also some representations of departure from the regular distribution, and has been the one hitherto used in acquiring the knowledge of the variations to which the vessels are subject. Much labour and research are conspicuous in its pages, and its publication must always ensure that commendation which an original work deserves. It consists of delineations of the anatomical distribution of the vessels according to the systematic description, and cannot be compared, for utility, with the present more extensive inquiries of Professor Quain. In the plates of Tiedemann more attention seems to be given to the small branches than to the large trunks, these being represented as mere cords without accompanying veins or nerves, which renders them almostvalueless for practical purposes. In those of Quain, on the contrary, the depth and distance are most exactly kept; and the accompanying veins and nerves with their variations, also the deviations in the form and extent of the neighbouring muscles, are always delineated so as to supply to the surgeon, at a glance, a just conception of the influence these may have in complicating the connexions of an arterial trunk. Tiedemann examined 500 bodies, and the plates are occupied with both the usual and unusual distribution, without any practical application, except in the single instance of aneurism from venesection. Quain has, as formerly noticed, observed 1040 bodies, "attention being always directed to those vessels, and to the points in their history, which seemed to be of importance in the practice of surgery." Although Tiedemann figures exceptions to the common branching of the vessels, he does not state how often each instance occurs. Quain not only enumerates the order of frequency with which any particular arrangement of the trunk of the vessel had been found, but gives tabular abstracts to assist the surgeon in deciding the probability of collateral or occasional branches interfering with the obliteration by the ligature. In the execution of the designs of the two there can scarcely be any comparison; those of Tiedemann are sometimes inaccurate, and appear rather plans than copies of nature; * whilst the figures of Quain are accurate and beautiful, and as truthful representations of the human body as any with which we are acquainted. The 'Anatomy and Operative Surgery of the

* As we have alluded to inaccuracies in the work of Tiedemann, it is but just to point to some. Seeing the labour bestowed on reference to authority, we cannot but be surprised at the negligence pointed out by Mr. Quain in the two following instances:

In fig. 8 of plate ix is a drawing of a variety of the arch of the aorta, observed by Zagorsky, a Russian anatomist. On referring to the original essay there is not any drawing, nor is there mention made of the state of the aorta, whether this was directed to the right or the left side.

Again, Meckel has misquoted Huber's observations respecting the origin of a thyroid artery from the arch of the aorta, and Tiedemann follows in the same error.

The following are some anatomical errors in the plates.

In plates ix and x the occipital artery is drawn as superficial to the splenius muscle instead of beneath it.

Plate xvi represents the median branch of the ulnar artery as being over (it should be under) the annular ligament in its course to the hand.

In plate xxiv the inferior mesenteric artery is improperly delineated between two layers of the peritoneum, which extend from the aorta to the great intestine; and the vessel is made to end in small branches to the sigmoid flexure of the colon, but every anatomist knows that it descends into the pelvis as a large branch (superior hemorrhoidal) to the rectum.

In plate xxvii the epigastric artery is superficial to the tendon of the transverse muscle in the lower part of the abdominal wall, whilst it should be beneath both the muscle and the fascia transversalis.

Arteries' of Professor Quain must not then be thought to be a work altogether similar to that of the Heidelberg Professor. True is it that in both the arteries are described. The work of Quain differs essentially in being almost altogether practical, and bestowing most attention on those dispositions of the larger trunks and branches which are concerned in operations, and have occasioned difficulties to the surgeon. Since the appearance of the English work the German publication must cease to be considered the measure of our knowledge; for the latter is less extensive in observations, less accurate in anatomy, and less practical in its nature than Quain's 'Anatomy of the Arteries.'

But we do not confine our approbation to the fact of the work containing what Tiedemann does not supply: it has peculiar merits of its own which entitle all engaged in the publication to great praise. It is one of those undertakings which necessarily occupy many years in accomplishing, and are not often attempted, from the time, expense, and labour required in their execution. It is indeed a national honour to have originated and executed a work that will be handed down to posterity, with those of Haller, Scarpa, Hunter, Sœmmerring, &c. as one alike laborious in research, correct in its representation of nature, and worthy to be imitated. In conclusion, it only remains for us to express our anxiety that the facts collected by Professor Quain should be generally known by the wide circulation of his work. Our conviction is, that no one who peruses it will fail to augment greatly his knowledge of the vascular system. To every operating surgeon and every anatomist its possession is essential; and into all public libraries it must command admission as the unquestioned standard authority on the anatomy and operative surgery of the arteries of the human body.

---

